# Cell Injection Millirobot Development and Evaluation in Microfluidic Chip

**DOI:** 10.3390/mi9110590

**Published:** 2018-11-13

**Authors:** Lin Feng, Qiang Zhou, Bin Song, Yanmin Feng, Jun Cai, Yonggang Jiang, Deyuan Zhang

**Affiliations:** 1 School of Mechanical Engineering & Automation, Beihang University, Beijing 100191, China; linfeng@buaa.edu.cn (L.F.); zhqiang1996@163.com (Q.Z.); songb@buaa.edu.cn (B.S.); feng_yan_min202@126.com (Y.F.); jun_cai@buaa.edu.cn (J.C.); zhangdy@buaa.edu.cn (D.Z.); 2 Beijing Advanced Innovation Center for Biomedical Engineering, Beihang University, Beijing 100083, China

**Keywords:** millirobot, injection, cell nucleus, magnetic control, cell surgery

## Abstract

We propose an innovative design of millirobot, which can achieve donor cell suction, delivery, and injection in a mammalian oocyte on a microfluidic chip. The millirobot body contains a hollow space that produces suction and ejection forces for the injection of cell nuclei using a nozzle at the tip of the robot. Specifically, a controller changes the hollow volume by balancing the magnetic and elastic forces of a membrane along with the motion of stages in the XY plane. A glass capillary attached to the tip of the robot contains a nozzle that is able to absorb and inject cell nuclei. The millirobot provides three degrees of freedom and generates micronewton forces. We demonstrate the effectiveness of the proposed millirobot through an experiment of the absorption and ejection of 20-µm particles from the nozzle using magnetic control in a microfluidic chip.

## 1. Introduction

Recent advances in biomedical engineering, particularly the manipulation of bioparticles (e.g., cells and biological tissue) in three-dimensional space, has attracted considerable attention in research and industry [1]. In particular, cell microsurgery is a new technology that uses modern research tools to perform certain operations on individual cells or groups of cells. Numerous studies have focused on microelectromechanical system technologies designed to manipulate and/or measure microorganisms [2]. In the 1960s, Berns proposed laser cell microsurgery through studies on cell genetics and kinematics. In 1972, Diacumakos and Tatum [3] used this technology for cell fusion. In 2011, Masaya Hagiwara et al. presented an innovative driving method for an on-chip robot actuated by permanent magnets in a microfluidic chip. A piezoelectric ceramic was applied to induce ultrasonic vibration in the microfluidic chip, and high-frequency vibration significantly reduced the effective friction on a magnetically driven microtool (MMT) [4]. In 2013, Feng et al. [5,6] achieved the on-chip enucleation of bovine oocytes using microrobot-assisted flow-speed control and constructed an accurate dispensing system for single oocytes. In addition, they achieved microparticle manipulations by utilizing a microrobot with passive diamagnetic levitation and noncontact cell transportation through the on-chip microfluid induced by oscillation of the microrobot [7,8]. Microfluidic cell cultures are ideally positioned to become the next generation of in vitro diagnostic tools for biomedical research [9].

Cell manipulation in the confined space of a microfluidic chip is highly important for biotechnology research because of low contamination risk and high repeatability and throughput, which make it an ideal medium to conduct experiments [10]. Microfluidic devices are highly modular, thereby providing the capacity to perform multiple functions [11]. In 2012, a multichannel microfluidic chip integrated with a novel graphene oxide (GO)-based Forster resonance energy transfer biosensor was created for the in situ detection of human acute lymphatic leukemia cells by assaying the cell-induced fluorescence recovery from GO/fluorophore carboxy fluorescein (FAM)-labeled aptamer oligonucleotides (GO/FAM-Sgc8) [12]. A microfluidic cell-culture chip, which was fabricated using a simple hybrid of the SU-8 and polydimethylsiloxane (PDMS) approach, was presented for trapping, cultivation, and releasing selected individual cells [13]. Stefan A. Kobel et al. [14] reported a reliable strategy to perform the automated image cytometry of single (nonadherent) stem cells captured in microfluidic traps. Despite recent technological advances, the development of a single device capable of simultaneously achieving high throughput, high target cancer cell recovery, high purity, and high cell viability remains challenging [15]. In 2013, Song-Bin Huang et al. [16] developed an integrated microfluidic cell culture system for high-throughput, microscale perfusion, three-dimensional, cell-culture-based assays. Furthermore, a microfluidic chip for the continuous-flow isolation and sorting of white blood cells from whole blood with high throughput and separation efficiency was reported in 2014 [17].

The robotic operation in a microfluidic chip outperforms human manipulation in the treatment of biological cells because of the high throughput and repeatability of such systems. The robotic control and power at this scale can be suitably achieved using magnetic fields that provide noncontact driving and consequently low invasiveness with respect to a cell, as well as low production costs. Several studies have been carried out on magnetic actuators [18,19,20,21,22]. For instance, an MMT with a permanent magnet can deliver a force of the order of millinewtons and ensure a small-sized driving unit. In fact, the magnetic field of a permanent magnet is more than ten times stronger than that of an electromagnetic coil of the same size [23]. Therefore, permanent-magnet actuation can be applied to a wide range of cell manipulation tasks, such as loading, sorting, and droplet generation [24,25,26]. Feng et al. [27] proposed an innovative driving method for a microrobot. They achieved a positioning accuracy of less than 1 mm using acoustic levitation, and response speed and output forces were significantly improved. A microrobot that manipulated balanced magnetic and buoyancy forces was fabricated in 2018 [28].

**Figure 1:**
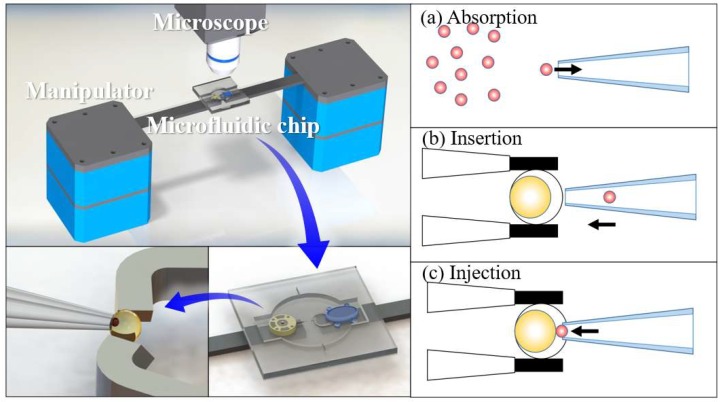
Millirobot system and process to inject cell nucleus into recipient cell.

In this study, we designed a robot for cell nuclei injection based on conventional methods. Figure 1 illustrates the proposed millirobot system and the actions required for the robot to inject a cell nucleus into a recipient cell. First, suction is produced at the tip of a glass capillary to catch a cell nucleus. Then, the robot moves to insert the tip of the glass capillary into a recipient cell. Finally, the nucleus is injected into the recipient cell.

**Figure 2:**
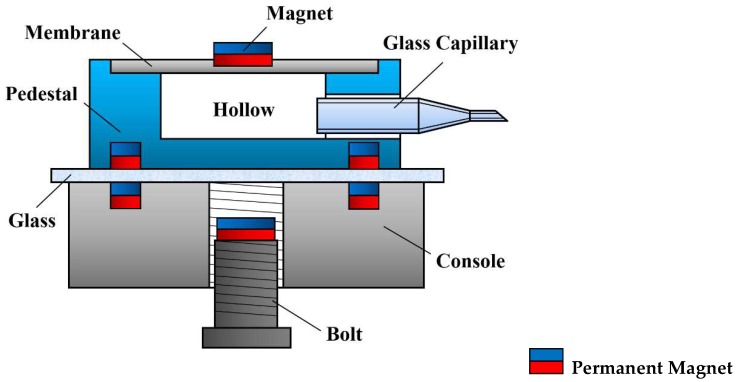
Structure of proposed millirobot.

Successful cell nucleus injection mostly depends on the absorption and injection operations. Figure 2 shows the structure of the proposed millirobot, whose tip is equipped with a glass capillary to accurately perform cell nucleus injection. A hollow covered with an elastic membrane is attached to the glass capillary, and a permanent magnet is fixed above the hollow. When the permanent magnet is moved downwards, the membrane is subjected to pressure, resulting in a depression that generates pressure inside the hollow. Consequently, ejection force is generated at the glass capillary tip for cell nucleus injection. On the contrary, when pressure is released by moving the permanent magnet upwards, the membrane bounces back, thus returning to its original shape and reducing the pressure inside the hollow. In this case, suction force is generated at the glass capillary tip for cell nucleus absorption. The movement of the permanent magnet is controlled by another permanent magnet beneath the chip, which moves up and down by fastening with a bolt. In addition, four other permanent magnets are uniformly distributed along the base of the robot to achieve arbitrary movements on the XY plane.

## 2. Materials and Methods

### 2.1. Membrane Deformation

The elastic membrane of the proposed millirobot enables the injection task to be performed. Therefore, the membrane deformation for different sizes of the membrane and magnet should be determined prior to the design of the other components. In this manner, the suitable dimensions of the hollow and the permanent magnet above it can be established. Ichikawa and Arai [29] compared and analyzed the deformation degree of SU-8 and PDMS membranes with thicknesses of 30, 50, 100, and 150 μm through simulations. They concluded that a PDMS elastic film with a thickness of 100 μm is the most suitable for their suction mechanism.

**Figure 3:**
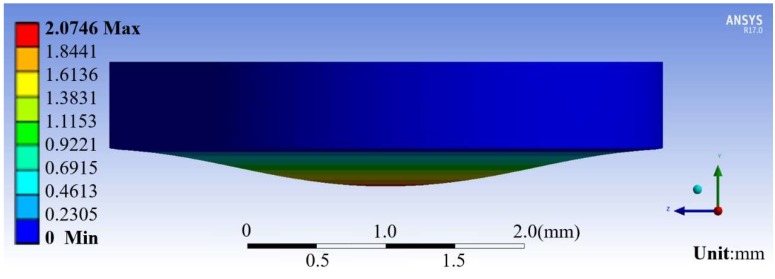
Simulated deformation of Ecoflex membrane caused by a permanent magnet (magnet diameter *d* = 2 mm, hollow diameter *D* = 4 mm).

However, we found that the PDMS membrane with a thickness of 100 μm was not sufficiently effective for our experiment. For example, it was breakable, and it was difficult to fix the magnet onto its surface. Therefore, we replaced the PDMS membrane with Ecoflex (Ecoflex™ 00-10, Smooth-On, Inc., Macungie, PA, USA). We simulated the deformation of a 600-µm thick Ecoflex membrane for different dimensions of the permanent magnets (thickness of 0.5 mm, diameters of 1 and 2 mm) and hollows (diameter of 3 and 4 mm). We compared the deformation under the action of gravity and under the influence of the permanent magnets. We used the ANSYS software (Ansys, Inc., Canonsburg, PA, USA) to simulate the deformation of the thin membrane when the permanent magnets were placed above the membrane without the influence of any other magnetic force. Then, the magnetic force between the permanent magnets above and below the membrane (Figure 2) was calculated according to the distance between the upper surface of the membrane and the lower surface of the glass plate, i.e., the distance between the two permanent magnets. Next, assuming that the force applied to the membrane was constant, we simulated the deformation of the membrane under magnetic force. The simulated model was simplified for estimating the minimum value of membrane deformation. Finally, by comparing the deformation with and without external magnetic force, we selected the combination of permanent magnet and hollow diameters that produced sufficient deformation for cell nucleus manipulation. The simulation results are shown in Figure 3 and summarized in Table 1.

As expected, the deformation of the membrane is the largest for the permanent magnet with a diameter of 2 mm and the hollow with a diameter of 4 mm. Under the influence of only gravity, the magnet at the center of the film moves downward by approximately 0.007 mm. Once magnetic force is added, the membrane and magnet move downward by approximately 2.075 mm, thus obtaining a deformation difference of approximately 2.068 mm. This is larger than the height of the hollow and thus enables it to produce sufficient suction for cell nucleus manipulation. Therefore, we selected the permanent magnet with a diameter of 2 mm and a thickness of 0.5 mm and the hollow with a diameter of 4 mm for the proposed millirobot.

### 2.2. Suction Principle

**Figure 4:**
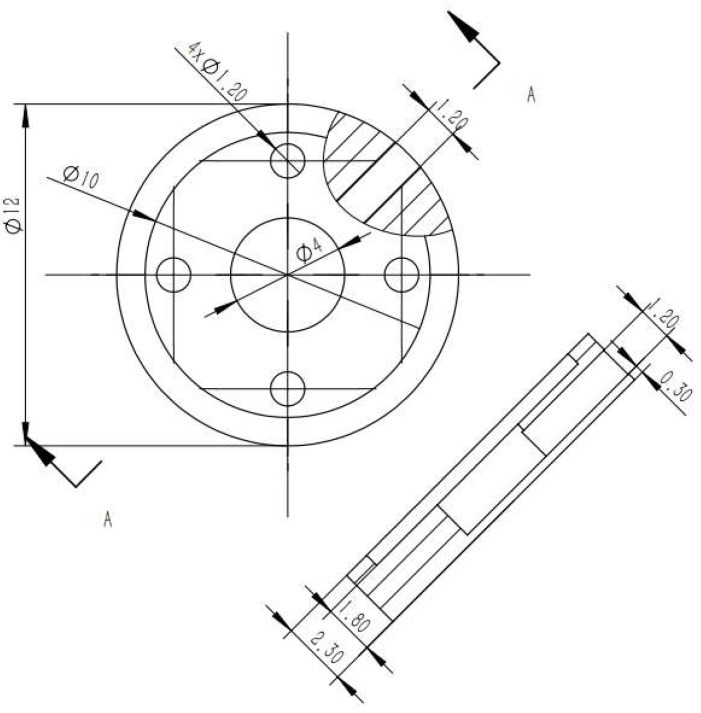
Diagram and dimensions of millirobot pedestal in millimeters.

We designed the other robot components after determining the diameters of the permanent magnet and hollow. The dimensions of the robot pedestal are shown in Figure 4.

**Figure 5:**
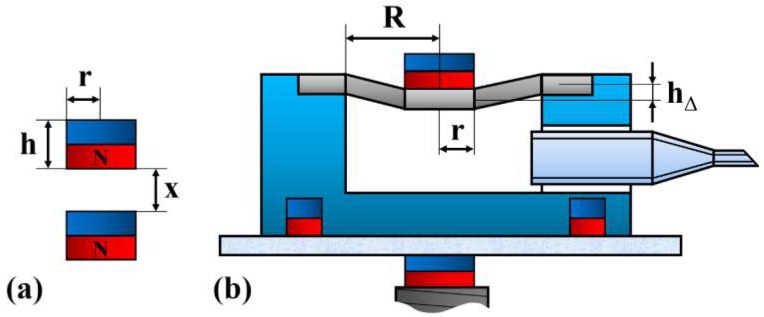
Diagram of employed permanent magnets and millirobot pedestal. (**a**) Parameters to determine the magnetic force between two cylindrical permanent magnets, (**b**) parameters to determine the hollow volume variation.

Considering the pedestal design, we derive the absorption and injection capabilities of the robot. The magnetic force between two cylindrical permanent magnets can be approximated as [30]

(1) $$\begin{array}{cc} F & =\frac{\pi \mu_{0}}{4}M^{2}r^{4}\left[\frac{1}{x^{2}}+\frac{1}{{\left(x+2h\right)}^{2}}-\frac{2}{{\left(x+h\right)}^{2}}\right]\\ & =\frac{\pi \mu_{0}}{4}{\left(\frac{B_{0}}{\mu_{0}}\right)}^{2}r^{4}\left[\frac{1}{x^{2}}+\frac{1}{{\left(x+2h\right)}^{2}}-\frac{2}{{\left(x+h\right)}^{2}}\right]\\ & =2.3125\;\mathrm{mN} \end{array}$$

where *r* is the radius of the cylindrical magnets, *h* is their thickness, *M* is their magnetization, *x* is the distance between them, as depicted in Figure 5a, *B*_0_ is the surface magnetic induction intensity, and *μ*_0_ is the vacuum magnetic permeability.

The membrane deforms under the magnetic force of the permanent magnets and gravity. As the membrane deformation under only gravity is negligible, the hollow volume variation, *Q*, can be approximated as a circular platform, as illustrated in Figure 5b, where *h*_Δ_ is the membrane deformation (~1.4 mm) and *R* is the radius of the cylindrical hollow. The hollow volume variation can be expressed as

(2) $$Q=V_{\Delta }=\frac{1}{3}\pi \cdot h_{\Delta }\cdot (R^{2}+r^{2}+R\cdot r)=9.81245\times 10^{-9}\;m^{3}$$

which enables us to calculate the suction force at the glass capillary tip. The diameter of the glass capillary tip on the millirobot is 25 μm, from which we calculate cross-sectional area *A*. In addition, we experimentally obtain an elastic membrane spring back time, *t*, of approximately 2 s after removing the magnetic force. During this period, the hollow volume varies by *Q* at an average flow velocity of *v* in the nozzle. According to Bernoulli’s equation for calculating pressure, we determine the resultant suction force, *F*, in the nozzle as follows:

(3) $$A={\left(\frac{d}{2}\right)}^{2}\cdot \pi =4.91\times 10^{-10}\;m^{2},$$

$$Q=9.81245\times 10^{-9}\;m^{3},$$

(4) $$v=\frac{1}{A}\cdot \frac{dQ}{dt}=9.9923\;m/s,$$

$$\rho =995.76\;\mathrm{kg}/m^{3},$$

(5) $$P=\frac{1}{2}\rho \cdot v^{2}=49711.5\;\mathrm{Pa},$$

(6) F=P⋅A=24.408 μN.

Then, we calculate the fluid resistance, *F*_s_, of the nucleus as it moves through the liquid. If this resistance is smaller than the suction force, suction is theoretically feasible for the proposed millirobot. Consider that the nucleus of a pig oocyte can be approximated as a sphere with a diameter of 20 μm. Using Stokes’ formula, we approximately calculate the motion resistance of this spherical nucleus in the cell liquid. Let *η* be the drag coefficient, *ɑ* be the radius of the sphere, and *v* be the velocity of the sphere in the fluid. In the considered case, *ɑ* = 10^–5^ m and *η* = 10^–3^ Pa·s. Given that the velocity of the sphere cannot be easily measured, we consider it to be the same as the fluid velocity through the nozzle, i.e., *v*_smax_ = *v* = 9.9923 m/s. As the actual velocity of the sphere must be below this value, the calculated resistance is the maximum. The suction force being higher than this maximum value confirms the feasibility of the proposed design.

(7) $$F_{s\max}=6\pi \eta av_{s}=1.8835\;\mu N<F.$$

### 2.3. Millirobot Fabrication

**Figure 6:**
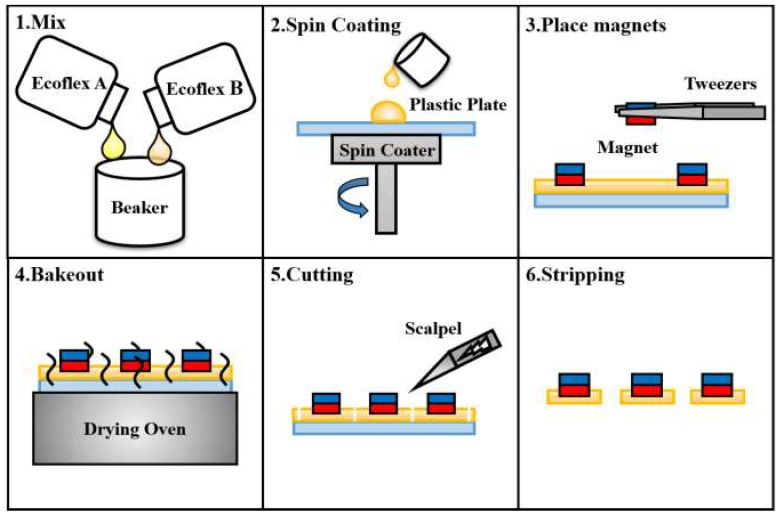
Fabrication process of the membrane for absorption/injection of the cell nucleus.

**Figure 7:**
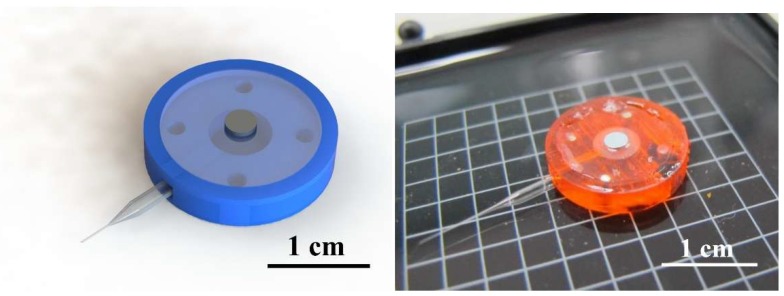
Concept drawing and photograph of the fabricated millirobot.

The proposed millirobot consists of three main components, i.e., the pedestal, membrane, and glass capillary. We fabricated the pedestal from a photosensitive resin through three-dimensional printing and the glass capillary using a drawing instrument. Figure 6 illustrates the membrane fabrication process. Ecoflex (Ecoflex™ 00-10) was used as the material for the membrane to achieve sufficient deformation. Liquid Ecoflex with a thickness of 600 μm was obtained for the membrane via spinning. The permanent magnets were placed on Ecoflex before it was fully solidified. After solidification, we cut a circular membrane with a diameter of 10 mm, ensuring that the permanent magnet was at the center of the membrane. The three components of the millirobot were integrated using the photosensitive resin as an adhesive (UV curable resin). The resulting robot is shown in Figure 7.

## 3. Experimental Setup

**Figure 8:**
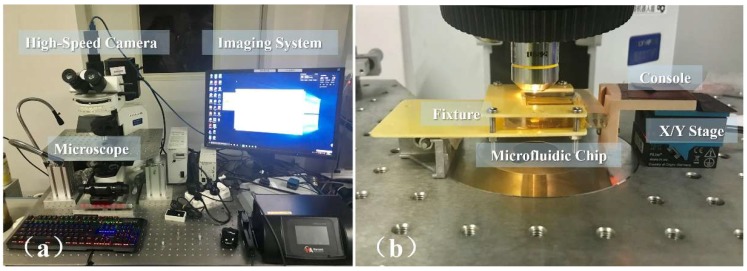
Photograph of experimental setup: (**a**) Observation system, (**b**) control system.

We tested the proposed millirobot for the operations of XY-plane motion, absorption, and ejection. Figure 8 shows the manipulation system and experimental setup consisting of control and observation systems. For the enhanced manipulation and visualization of the microfluidic chip, the experiment was conducted under a microscope (CX41; Olympus Co., Tokyo, Japan) with a mounted camera (GS3-U3-23S6C-C; FLIR Integrated Imaging Solutions, Ltd., Richmond, BC, Canada) and external illumination. The microfluidic chip was fixed on a stage, and the manipulator was operated with a computer joystick. The equipment was fixed to a shockproof platform to ensure high positioning accuracy.

A stage with two degrees of freedom (U-521 PILine^®^ XY Stage, Physik Instrumente GmbH & Co., Karlsruhe, Germany) was used in the experiment. The stage had a precision of 300 nm, which was suitable for the size of the bioparticles and millirobot. We used a PDMS microfluidic chip (Dow Corning Co., Midland, MI, USA) filled with water and a glass substrate as the experimental platform. The robot and bioparticles, which were represented by polystyrene microbeads with a diameter of 20 μm, were packed within the microfluidic chip to prevent contamination. The channel of the microfluidic chip was designed specifically for testing the robot.

## 4. Experimental Results

**Figure 9:**
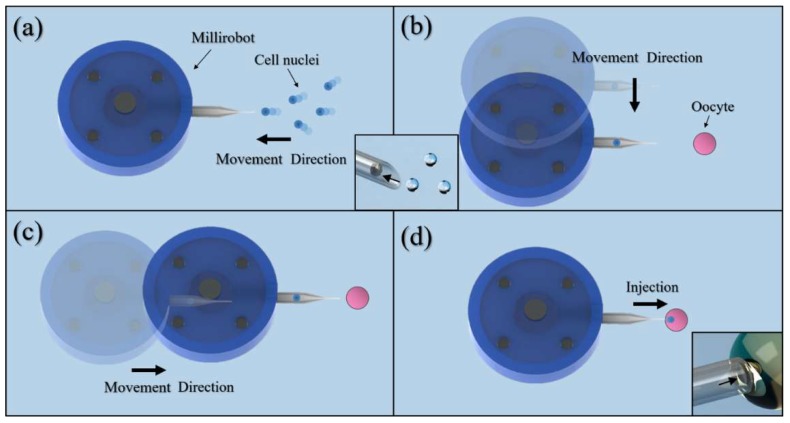
Concept of experimental process. (**a**) The process of cell nuclei absorption; (**b**) Millirobot motion along the Y axis; (**c**) Millirobot motion along the X axis; (**d**) The injection of cell nuclei. Concept of experimental process. (**a**) The process of cell nuclei absorption; (**b**) Millirobot motion along the Y axis; (**c**) Millirobot motion along the X axis; (**d**) The injection of cell nuclei.

**Figure 10:**
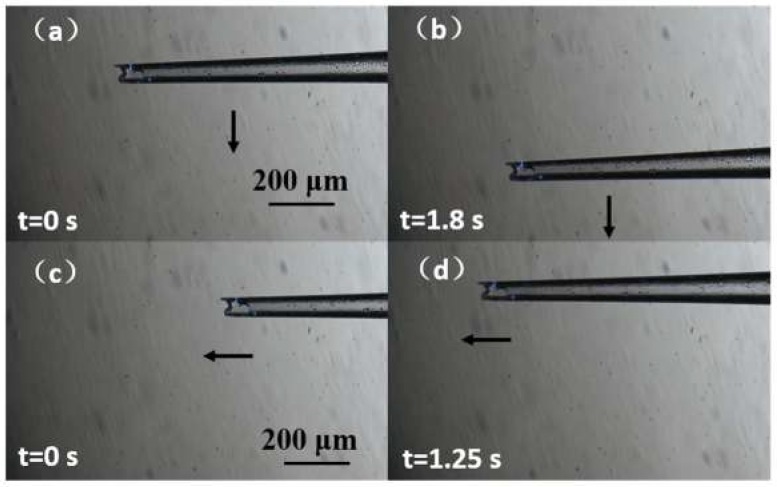
Millirobot motion along (**a**,**b**) the Y axis and (**c**,**d**) X axis.

**Figure 11:**
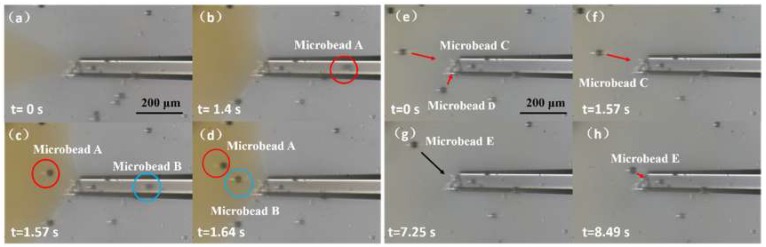
(**a**–**d**) Microbead ejection from the nozzle. (**e**–**h**) Microbead absorption on a glass capillary.

Figure 9 shows the concept of the experimental process. In the actual experiment, we used microbeads to replace the cell nuclei and simplified the injection to ejection. First, we tested the XY-plane motion control of the millirobot. This was achieved by operating the software to control the stage. The sequences of motions along the X and Y axes are shown in Figure 10 (Supplementary videos 1–4). Then, we evaluated the absorption and ejection capabilities of the proposed membrane mechanism over polystyrene microbeads. Figure 11 shows the ejection sequence of two microbeads by controlling the concave deformation of the membrane. Microbead A leaves the glass capillary first, followed by microbead B after waiting for ejection. Similarly, Figure 11 shows the sequence of microbead absorption. The microbead trajectories during absorption are indicated by arrows. Absorption is accomplished by removing the magnetic force and letting the membrane spring back (Supplementary videos 5 and 6).

**Figure 12:**
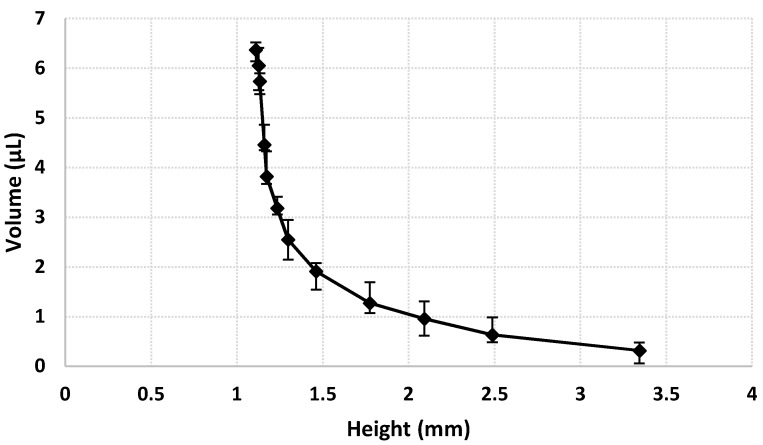
Relation between hollow volume and distance between the permanent magnet and the microfluidic chip during particle ejection.

We confirmed the absorption and ejection capabilities of the millirobot through the abovementioned experiments. During these experiments, the movement distance of the magnet under the chip was directly related to the hollow volume variation, as shown in Figure 12 for microbead ejection, where the hollow volume variation is plotted according to the distance between the magnet and the bottom of the chip. The volume increases exponentially as the magnet and the bottom of the chip approach each other. Volume *V* indicates the amount of liquid that is sprayed out of the millirobot.

## 5. Conclusions

We propose a method for the two-dimensional control of a millirobot within a microfluidic chip aimed for cell surgery. The millirobot contains a hollow space to produce suction and ejection forces that can be used for the injection of a cell nucleus using a nozzle at the tip of the robot. We experimentally confirm the absorption and ejection capabilities of the proposed mechanism using polystyrene microbeads with a diameter of 20 μm. In the future, we will conduct experiments using an actual cell nucleus and complete the process of injecting it within a recipient cell. In addition, as the relationship between magnetic force, the distance between permanent magnets, and the hollow volume variation is nonlinear and difficult to control, we plan to replace the permanent magnet at the bottom of the chip with an electromagnetic coil to improve accuracy and controllability during the absorption and ejection operations.

## Figures and Tables

**Table 1:** Results of simulated deformation of Ecoflex membrane caused by a permanent magnet.

Magnet Diameter *d* (mm)	Hollow Diameter *D* (mm)	Deformation under Gravity (mm)	Deformation under Magnetic Force (mm)	Deformation Difference (mm)
1.0	3.0	0.001485	0.117260	0.115775
2.0	3.0	0.003538	1.106800	1.103261
1.0	4.0	0.002335	0.184310	0.181975
2.0	4.0	0.006633	2.074600	2.067967

## Supplementary Materials

The following are available online at http://www.mdpi.com/2072-666X/9/11/590/s1, Video 1: [Macroscopic] Microrobot motion along the X axis, Video 2: [Macroscopic] Microrobot motion along the Y axis, Video 3: Microrobot motion along the X axis, Video 4: Microrobot motion along the Y axis, Video 5: Microbead absorption to glass capillary, Video 6: Microbead ejection from nozzle.
